# Design of Non-Haemolytic Nanoemulsions for Intravenous Administration of Hydrophobic APIs

**DOI:** 10.3390/pharmaceutics12121141

**Published:** 2020-11-25

**Authors:** Line Séguy, Anne-Claire Groo, Didier Goux, Didier Hennequin, Aurélie Malzert-Fréon

**Affiliations:** 1 Centre d’Etudes et de Recherche sur le Médicament de Normandie (CERMN), UniCaen, Normandie University, 14000 Caen, France; line.seguy@unicaen.fr; 2 Centre de Microscopie Appliquée à la biologie (CMAbio), UniCaen, Normandie University, SF4206 Icore, 14000 Caen, France; didier.goux@unicaen.fr; 3 UR Aliments Bioprocédés Toxicologie Environnements (ABTE), UniCaen, Normandie University, 14000 Caen, France; didier.hennequin@unicaen.fr

**Keywords:** nanomedicine, self-emulsification, safety profile, haemolysis assay, preclinical studies

## Abstract

Among advanced formulation strategies, nanoemulsions are considered useful drug-delivery systems allowing to improve the solubility and the bioavailability of lipophilic drugs. To select safe excipients for nanoemulsion formulation and to discard any haemolytic potential, an in vitro miniaturized test was performed on human whole blood. From haemolysis results obtained on eighteen of the most commonly used excipients, a medium chain triglyceride, a surfactant, and a solubilizer were selected for formulation assays. Based on a design of experiments and a ternary diagram, the feasibility of nanoemulsions was determined. The composition was defined to produce monodisperse nanodroplets with a diameter of either 50 or 120 nm, and their physicochemical properties were optimized to be suitable for intravenous administration. These nanoemulsions, stable over 21 days in storage conditions, were shown to be able to encapsulate with high encapsulation efficiency and high drug loading, up to 16% (*w*/*w*), two water practically insoluble drug models: ibuprofen and fenofibrate. Both drugs may be released according to a modulable profile in sink conditions. Such nanoemulsions appear as a very promising and attractive strategy for the efficient early preclinical development of hydrophobic drugs.

## 1. Introduction

Since the liposomal formulation called Doxil^®^ in 1995, nanomedicine development progressed and the number of nanodrugs, approved by the Food and Drug Administration (FDA), has increased markedly [1]. Many types of nanoparticles administered by various administration routes were brought into commercial products [2,3], for instance aprepitant (Emend^®^) by oral administration, amphotericin B (Ambisome^®^) by intravenous (IV) route, or leuprolide acetate (Eligard^®^) by the subcutaneous one [4,5,6]. The interest of nanomedicines is clearly established to permit the therapeutic valorisation of drugs [7,8], new or in a repurposing approach [9,10]. It can be also considered in the drug-discovery step to permit preclinical studies of leads [1]. Indeed, the advent of modern drug-discovery methods, such as high-throughput screening or fragment-based drug design, has contributed to the identification of lead candidates with increased selectivity and potency [11,12]. However, these compounds were obtained at the expense of other properties, as reflected by their higher lipophilicity and molecular weight [11,12]. Consequently, such changes negatively impact their solubility and their permeability and, then, can limit their bioavailability and their therapeutic effect [13,14]. Thus, more than half of new chemical entities in drug-development pipeline are practically insoluble in water [15,16]. Due to these poor intrinsic properties, these molecules require the use of formulation strategies to reach preclinical and clinical studies. Therefore, advanced formulation methods must be considered as soon as possible in drug discovery process, and lipid-based nanoformulations, such as lipid nanoemulsions (NE), may offer valuable solutions [17].

Ideally, a nanoformulation should be as versatile as possible, i.e., suitable to various drugs and to many routes of administration. Although oral administration route is the most common route in animal dosing [18], parenteral routes are widely used in early pharmacology, pharmacokinetics, and toxicology studies [18,19]. Among them, the IV administration route is the most efficient but also the most restrictive one in terms of requirements [18,20]. In accordance with the pharmacopeia and as reported in the literature, intravenous formulations must be sterile, particulate-free, and isotonic, within pH 2–9 preferably and their haemolytic potential must be evaluated [21,22,23]. Indeed, red blood cell lysis by surfactants is a well-known phenomenon [24,25]. It can lead to anaemia and even immediate death when the haemolytic potential of excipients administered intravenously is very high [26]. The integration of the biocompatibility assessment of each excipient and of nanoformulations with blood components during early preclinical development seems essential. Knowing their erythrocyte-damaging potential, the choice of excipients can be optimized in formulation development. Various haemolysis protocols for nanoformulations are reported in the literature. In order to be more predictive, this assay have to be realized on human whole blood [22,27] although these assays are also executed on human [28], rat [29], or sheep [30] red blood cells in the literature.

The aim of the present work is to develop a nanoemulsion formulation on the basis of the biocompatibility profile of its excipients determined through a valuable miniaturized process using human whole blood. Then, its interest for the delivery of two water practically insoluble active pharmaceutical ingredients (APIs) will be evaluated. Nanoemulsions were generated at room temperature by a spontaneous emulsification process. Two formulations were obtained, with different droplet sizes. Ibuprofen- and fenofibrate-loaded nanoemulsions were formulated based on these two formulations. Their physicochemical properties were characterized in terms of size, polydispersity, surface potential, drug-recovery efficiency, pH, osmolarity, in vitro drug release, and stability in storage conditions and in biomimetic medium to evaluate their potential for IV administration.

## 2. Materials and Methods

### 2.1. Materials

Kolliphor^®^ EL (macrogolglycerol ricinoleate), Kolliphor^®^ HS15 (macrogol 15 hydroxystearate: 70% polyethylene glycol (PEG) 660 hydroxystearate and 30% free PEG 660), Kollisolv^®^ PG (propylene glycol), and Kollisolv^®^ PEG 400 (macrogol) were kindly provided by BASF (Ludwigshafen, Germany). Labrafac^®^ WL 1349 (medium chain triglyceride), Maisine^®^ CC (glycerol monolinoleate), Peceol^®^ (glycerol mono-oleates, type 40), Plurol^®^ Oleique CC 497 (polyglyceryl-3 dioleate), Transcutol^®^ HP (diethylene glycol monoethyl ether), and the macrogolglycerides: Labrafil^®^ M 1944 (oleic), Labrafil^®^ M 2125 (linoleic), and Labrasol^®^ (caprylocapric) were gifts from Gattefossé S.A. (Saint-Priest, France). Lipoid^®^ E PC (egg yolk phosphatidylcholine), Lipoid^®^ S LPC 80 (soy lysophosphatidylcholine), and Lipoid^®^ S 100 (soy phosphatidylcholine) were generously provided by Lipoid GmbH (Ludwigshafen, Germany). Miglyol^®^ 812 (medium chain triglyceride) and Captex^®^ 200 (propylene glycol dicaprylate/dicaprate) were respectively kindly donated by IOI Oleochemical (Witten, Germany) and Abitec Corporation (Janesville, Wisconsin, USA). Due to the complex composition of the excipients, the brand names are used throughout the text. KH_2_PO_4_, Na_2_HPO_4_·12 H_2_O, NaH_2_PO_4_·H_2_O, Brij^®^ L23 (polyoxyethylene (23) lauryl ether), Tween^®^ 80 (polysorbate 80), human haemoglobin, Drabkin’s reagent, Dulbecco’s phosphate buffered saline (DPBS), and Triton^®^ X-100 (octylphenol PEG-10 ether) were obtained from Sigma-Aldrich (Steinheim, Germany). NaCl was provided by Acros Organics (Geel, Belgium). Ibuprofen and fenofibrate were purchased respectively from Fagron (Saint-Denis, France) and Sigma-Aldrich (Steinheim, Germany). Water, methanol, and acetonitrile of HPLC analytical grade were obtained from Fisher Scientific (Loughborough, United Kingdom). The demineralised water used was obtained using a mixed bed ion exchange resin, Distiplus DS450 (Grosseron, Couëron, France).

### 2.2. Methods

#### 2.2.1. Formulation of Nanoemulsions

Nanoemulsion formulation was adapted from the spontaneous nano-emulsification method previously described by our team [31]. The anhydrous phase, composed of oil (Labrafac^®^ WL 1349), surfactant (Kolliphor^®^ HS 15), and solubilizer (Transcutol^®^ HP), was heated at 70 °C under gentle magnetic stirring (250 rpm) and cooled down at 25 °C. When the anhydrous mixture reached this temperature, the magnetic stirring was increased from 250 rpm to 750 rpm and the aqueous phase (10 mM or 65 mM phosphate buffer, 25 °C) was suddenly added, leading to spontaneous emulsification. After the addition of water, stirring was maintained for 15 min at room temperature. Then, the formulation was filtered through 0.2-µm regenerated cellulose syringe filters (Minisart^®^ Syringe Filter, Sartorius, Goettingen, Germany). In case of API-loaded nanoemulsions, the API was weighed with the anhydrous phase, heated at 70 °C under gentle magnetic stirring (250 rpm) and mixed for 5 min by ultrasonic treatment at room temperature. Then, nanoemulsions were prepared as previously described.

#### 2.2.2. Physicochemical Characterization of the Nanoemulsions

Dynamic light scattering (DLS) was used to determine the average hydrodynamic diameter, the polydispersity index (PDI) and the diameter distribution by volume of the nanoemulsions using a NanoZS^®^ apparatus (Malvern Instruments, Worcestershire, UK) equipped with a 633 nm laser at a fixed scattering angle of 173°. The temperature of the cell was kept constant at 25 °C. The nanoemulsions were diluted 1/100 (*v*/*v*) in NaCl 1 mM in order to assure an appropriate scattered intensity on the detector before measurements. Measurements were performed in triplicate.

#### 2.2.3. Zeta Potential Measurement

Zeta potential analyses were realized, after filtration and 1/100 dilution in NaCl 1 mM, using a NanoZS^®^ apparatus equipped with DTS 1070 cell. All measurements were performed in triplicate at 25 °C, with a dielectric constant of 78.5, a refractive index of 1.33, a viscosity of 0.8872 cP and a cell voltage of 150 V. The zeta potential was calculated from the electrophoretic mobility using the Smoluchowski equation.

#### 2.2.4. pH and Osmolarity Measurements

The pH of nanoemulsions was measured using a pH-meter (Eutech instrument, Landsmeer, Netherlands) equipped with a microprobe (Fisherbrand, Fisher Scientific, Illkirch, France). The osmolarity of nanoemulsions was measured using a micro-osmometer autocal type 15/15M (Löser Messtechnik, Berlin, Germany) via freezing-point method. Typically, 100 µL of nanoemulsions were introduced in microtube and measurements were performed.

#### 2.2.5. Transmission Electronic Microscopy

The morphology of the nanoemulsions was examined under transmission electron microscopy (TEM) using a Jeol 1011 apparatus (Jeol, Japan) and a camera Orius 200 (Gatan, France). Before analysis, the nanoemulsions were 10-fold diluted with ultrapure water. Then, nanoemulsions were dropped on formvar grids previously cleaned by glow discharge (Elmo Cordouan Technologies, Pessac, France). Finally, the sample was shaded with a 1.5% uranyl acetate solution for fifteen seconds.

#### 2.2.6. Determination of the Encapsulation Efficiency and the Drug Loading

The encapsulation efficiency (EE) was determined after filtration through 0.2 µm syringe filters (Minisart^®^ Syringe Filter, Sartorius, Goettingen, Germany) to remove unentrapped APIs. Then, these samples were diluted in methanol (1/500, *v*/*v*) and the concentrations of ibuprofen and fenofibrate were determined by high-performance liquid chromatography (HPLC), as described in Section 2.2.9. The EE was determined in triplicate and calculated as follows:

(1) $$\mathrm{EE}\left(\%\right)=100\times \frac{\mathrm{Quantity}\;\mathrm{of}\;\mathrm{API}\;\mathrm{entrapped}}{\mathrm{Total}\;\mathrm{quantity}\;\mathrm{of}\;\mathrm{API}\;\mathrm{added}}$$

The drug loading (DL) was defined as follows:

(2) $$\mathrm{DL}\left(\%\right)=100\times \frac{\mathrm{Quantity}\;\mathrm{of}\;\mathrm{API}\;\mathrm{entrapped}}{\mathrm{Total}\;\mathrm{quantity}\;\mathrm{of}\;\mathrm{anhydrous}\;\mathrm{excipients}}$$

#### 2.2.7. Stability Studies

The short-term stability of the blank nanoemulsions was investigated over a storage period of 21 days both at room temperature (20 ± 2 °C) and at 4 °C. The stock formulations (without dilution to mimic storage conditions) were stored at 4 °C or 20 °C and diluted at regular intervals with a 1/100 (*v*/*v*) dilution in NaCl 1 mM for evaluating the size distribution and zeta potential. A stability study at 37 °C for 24 h was also accomplished to mimic operating conditions for future in vitro and in vivo studies. Nanoemulsions were diluted at 1/100 in phosphate buffered saline (PBS), pH 7.4 (European pharmacopeia, 9th ed.), and were then placed in tubes in a water bath WNB-22 (Memmert, Schwabach, Germany) at 37 °C under gentle horizontal shaking. The size measurements and distribution were performed just after dilution and after incubation. All assays and measurements were performed in triplicate.

#### 2.2.8. In Vitro API Release Kinetics Studies

The release of each API from nanoemulsions was studied by the dialysis bag method. One millilitre of API-loaded nanoemulsions was instilled into a cellulose ester dialysis bag (Spectra/Por^®^ Biotech membranes, molecular weight cutoff of 100 kDa, Spectrum Laboratories, Rancho Dominguez, CA, USA) and incubated in PBS, pH 7.4. The incubation was realized in a water bath WNB-22 at 37 °C under gentle horizontal shaking. Samples of 1 mL were withdrawn at appropriate intervals, and the same volume was replaced with fresh PBS. In case of fenofibrate, polysorbate 80 (1%, *v*/*v*) was added to the acceptor compartment to respect sink conditions. The percentage of API released was measured by HPLC by taking into account the cumulative quantity removed. All measurements were performed in triplicate.

The data obtained were fitted according to zero-order (Q = Q_0_ + k × t), first-order (ln Q = ln Q_0_ + k × t), and square root of time (Q = k × √t) models, where Q (mg) denotes the cumulative amount of drug released at time t (h), Q_0_ is the initial amount of drug at t = 0, and k is the release constant (Table 1). It can be noted that the Higuchi equation, describing diffusional release from a thin film, would be misused in the present study since various defining conditions are violated [32].

#### 2.2.9. HPLC Methods

Drugs concentrations were determined by HPLC by using methods previously reported by our team [31]. The HPLC system comprised an Agilent^®^ 1290 Infinity binary pump, an Agilent 1290 Infinity autosampler, and an Agilent 1260 Infinity diode-array detector (Agilent technologies, Santa Clara, CA, USA). A reversed phase column C18 (5 μm, 2.1 × 50 mm, Restek^®^ Ultra, Lisses France) was used as the analytical column. The mobile phase was composed of a mixture of acetonitrile containing 0.1% (*v*/*v*) formic acid (A) and water containing 0.1% (*v*/*v*) formic acid (B). Detection wavelength (λ), flow rate, total run time (T), gradient, injection volume, concentration range, and retention time (Rt) used to analyse ibuprofen and fenofibrate are listed in Table 2. The column temperature was 40 °C. Linearities were good within the concentration ranges studied with a correlation coefficient higher than 0.99. The detection limits were 2.08 μM and 2.16 μM, and the quantification limits were 6.31 μM and 6.55 μM for ibuprofen and fenofibrate, respectively. The detection and quantification limits were evaluated with the standard deviation of the response and the slope [33].

#### 2.2.10. In Vitro Haemolysis Assay

Haemolysis tests were adapted from the protocol initially described by Dobrovolskaia et al. [22]. Whole human blood samples from three healthy compatible volunteers were collected in Li-heparin tubes (Etablissement Français du Sang, EFS Hauts-de-France-Normandie, France). For overcoming any variability, the three samples were pooled, and the total haemoglobin concentration was measured and adjusted to 10 mg/mL by dilution with DPBS (SD_10mg/mL_). An aliquot (900 µL) of the pooled whole blood was centrifuged for 15 min at 3000 rpm to determine plasma-free haemoglobin (PFH).

The various excipients and nanoemulsions were assayed in the final concentration range 0.05–2.50 mg/mL. For each test, 100 μL of the excipient or nanoemulsion solutions was introduced into Eppendorf tubes with 700 μL of DPBS and 100 μL of SD_10mg/mL_ and incubated for three hours at 37 °C with constant horizontal shaking (water bath WNB-22, Memmert, Schwabach, Germany). After incubation, the samples were centrifuged (15 min at 3000 rpm, 25 °C using a Universal 320R apparatus, Hettich, Bäch, Switzerland) to separate the pellet containing undamaged erythrocytes from the supernatant containing the haemoglobin released during haemolysis. Haemolysis percentage was quantified by spectrophotometry (Infinite M200, Tecan, Männedorf, Switzerland) by determining the absorbance of red cyanmethaemoglobin (CMH) at 540 nm, at 25 °C, after the addition of Drabkin’s reagent (dissolved in deionized water in the presence of Brij^®^ L23 0.05% (*w*/*w*)) in a 96-well plate. These measured absorbances were compared to a standard curve of human haemoglobin with satisfactory linearity (R^2^ > 0.99) in the concentration range studied (0.0625–1 mg/mL). The concentration of haemoglobin in the supernatant was compared to those in the supernatant of a untreated blood sample with samples to obtain the percentage of the sample-induced haemolysis (referred to as percent haemolysis).

Before each experiment, PFH was determined and must not exceed 1% of the total haemoglobin. Each test was approved with a positive control—Triton^®^ X-100, known to be haemolytic—and with a negative control—PBS. In order to overcome potential particles, optical interferences with the signal emitted at or close to the assay wavelength (540 nm), false positives, and false negatives were realized for each test. The results shown for the nanoemulsions were adjusted taking into account for these interferences.

For each compound tested, haemolytic properties were evaluated according to the haemolysis percentage calculated as follows:

(3) $$\mathrm{Haemolysis}\left(\%\right)=100\times \frac{{\left[\mathrm{Cyanmethaemoglobin}\right]}_{\mathrm{supernatant}}}{{\left[\mathrm{Cyanmethaemoglobin}\right]}_{\mathrm{total}}}$$

In this assay, a haemolysis threshold of 5% was defined. When it was crossed, the compound was considered haemolytic. All assays were performed in triplicate.

#### 2.2.11. Mixture Experiments

**Figure 1:**
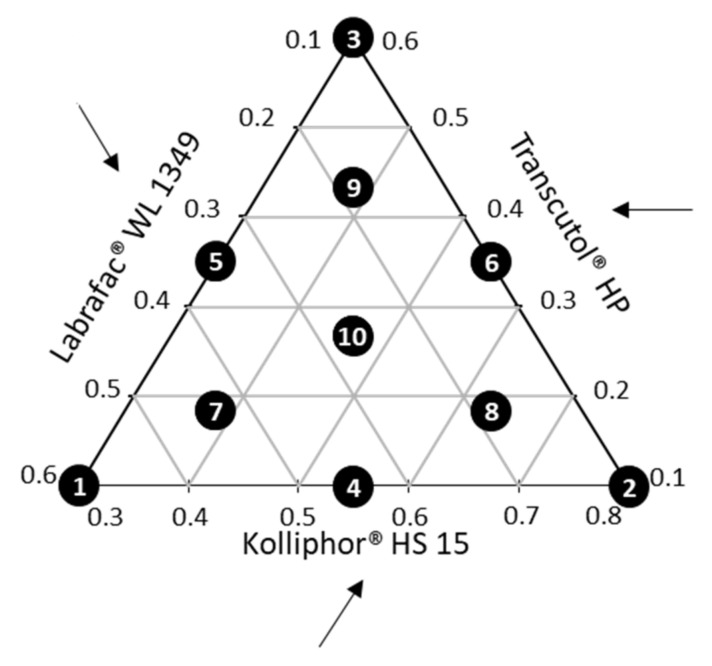
Ternary diagram for the mixture design: the central point (point 10) was triplicated.

A pseudoternary diagram was constructed to evaluate the nanoemulsions formation by modulating the proportions of the three excipients into their respective weight fraction defined according to our knowledge: 0.1 < Labrafac^®^ WL 1349 < 0.6, 0.3 < Kolliphor^®^ HS 15 < 0.8, and 0.1 < Transcutol^®^ HP < 0.6. The total amount of the 3 excipients was always 100% (*w*/*w*). Ten experiments were defined (Figure 1), and the barycentre, point 10, was realized in triplicate. A formulation was considered acceptable if the size of the particles was lower than 150 nm and the PDI was lower than 0.2.

#### 2.2.12. Partial Least Square (PLS) Analysis

An explanatory statistical analysis was executed to determine the influence of the components on the size and polydispersity of the nanoemulsions. Three variables X_1_, X_2_, and X_3_ were considered corresponding to the amount of Labrafac^®^ WL 1349, Kolliphor^®^ HS 15, and Transcutol^®^ HP, respectively. Two responses variables were taken into account, i.e., Y_1_: average diameter in nm and Y_2_: PDI. The data for diameter were log-transformed prior to analysing to fit a normal distribution. The PLS regression analysis was carried out with the MODDE V10 software (Sartorius Stedim Data Analytics AB Malmö, Sweden). The model used is a second-degree Cox polynomial adjusted by PLS regression:

(4) $$Y=b_{0}+\overset{k}{\underset{i=1}{\sum }}b_{i}X_{i}+\underset{i<j}{\sum }b_{\mathrm{ij}}X_{i}X_{j}+\overset{k}{\underset{i=1}{\sum }}b_{\mathrm{ii}}X_{i}^{2}$$

where Y is the model response; b_0_ is the constant of the model; b_i_, b_ij_, and b_ii_ are the regression coefficients; and X_i_, X_i_X_j_, and X_i_^2^ are the associated variables. The algorithm NIPALS (Nonlinear Iterative Partial Least Squares) was used to develop the PLS regression.

## 3. Results and Discussion

### 3.1. In Vitro Haemolysis Assay

By transposing the assay proposed by Dobrovolskaia et al. [22], initially conceived for the analysis of the haemolytic properties of nanoparticles, we performed a quantitative colorimetric determination of total haemoglobin in whole human blood and of plasma-free human haemoglobin in the presence of selected excipients. This miniaturized test is based on the colorimetric detection of red cyanmethaemoglobin in solution by spectrophotometry at its maximum absorbance (540 nm). As proposed in this protocol, excipients were assayed at 4 concentrations after dissolution/resuspension in PBS by considering a theoretical plasma concentration of X = 0.25 mg/mL, 10X (2.5 mg/mL), and two dilutions of this theoretical plasma concentration, i.e., 0.05 and 0.1 mg/mL. An increase in haemoglobin is indicative of erythrocyte damage by the tested compound. According to the results, materials with in vitro haemolysis values above 5% should be haemolytic in vivo and should be discarded for any IV administration.

Table 3 shows the results of haemolysis determined for 18 excipients which were rationally selected among lipid vehicles, surfactants, and solubilizers that are usually used for the formulation of nanoemulsions and more widely for nanoparticles. Some of them are already approved for parenteral use, e.g., Labrafac^®^ WL 1349, Miglyol^®^ 812, Lipoid^®^ E PC, Lipoid^®^ S 100, Kollisolv^®^ PEG 400, Kolliphor^®^ HS15, Kolliphor^®^ EL, Kollisolv^®^ PG, and Tween^®^ 80 [34]. Among the excipients not approved for IV route, the three macrogolglycerides are recommended for oral, topical, or vaginal routes, but given their interesting properties, they are reported in several formulation assays for IV administration [35,36,37]. Transcutol^®^ HP is approved by the FDA for topical and transdermal routes, and besides, the manufacturer recommends its use for parenteral route. They were included in this study.

The oily vehicles containing long-chains fatty acids, Maisine^®^ CC, Peceol^®^, and Plurol^®^ Oleique CC 497, could not be solubilized in PBS in spite of the various strategies employed (sonication or/and heating). Hence, their haemolytic properties could not be determined. An emulsion was formed when each lecithin was added to PBS, explaining the higher standard deviation. Phosphatidylcholine (PC) from egg yolk lecithin (Lipoid^®^ E PC) and from soybean lecithin (Lipoid^®^ S 100) appear quite safe regarding haemocompatibility. Our results are in agreement with those obtained by Bender et al. [38] about the haemocompatibility of some lipid-core nanocapsules stabilized with lecithin. Thus, such excipients are interesting to formulate liposomes-based nanoparticles or can be used as efficient emulsifying or stabilizing agents of nanoformulations to be administrated by the IV route. On the contrary, the soybean lysophosphatidylcholine (LPC) commercially named Lipoid^®^ S LPC 80 and containing 80.8% monoacyl phosphatidylcholine (MAPC) and 13.2% phosphatidylcholine appears highly haemolytic. With an haemolytic potential above 50%, it should cause immediate animal death when administered intravenously [26]. This is probably reliable to MAPC, a natural surfactant present in the human intestinal tract as a digestion product of phospholipids. This compound has been already proposed in the formulation of self-emulsifying drug delivery systems (SEDDS) as an alternative to synthetic surfactants to limit irritancy to cell membranes of the digestion tract and to possibly modify the digestion rate [39,40]. In our test, the lowest concentration assayed for LPC was 4-fold superior to its critical micellar concentration (CMC) [41]. By forming micelles, LPC would cause haemolysis [42,43]. Obviously, this natural emulsifier used in lipid-based formulations for oral administration cannot be used for intravenous administration.

The oily vehicles containing medium-chains fatty acids, Labrafac^®^ WL 1349 and Miglyol^®^ 812, were not haemolytic, even at the highest concentration tested. Moreover, possessing greater drug solubilization ability than long-chains fatty acids [44], they appear particularly appealing to formulate lipid-based formulations for IV route.

Surfactants with high hydrophilic-lipophilic balance (HLB) were chosen considering their ability to produce fine oil-in-water (O/W) nanoemulsions by low-energy methods. Kolliphor^®^ EL had no haemolytic activity whatever the concentration studied. However, regarding the hypersensitivity reactions due to its use and its neurotoxic potential [45,46], this excipient must be considered carefully in the development of versatile nanoemulsions. Tween^®^ 80 appeared haemolytic from a plasma concentration of 0.25 mg/mL. This is in agreement with studies reporting that concentration higher than 80 µL/mL for Tween^®^ 80 induces 50% haemolysis of the erythrocytes [47]. Our results indicate that only a diluted nanoformulation based on this surfactant could be envisaged but limits the versatility of the development. Concerning Kolliphor^®^ HS 15, its haemolytic activity was negligible at a plasma concentration of 0.25 mg/mL. It became significant only at the highest concentration tested of 2.5 mg/mL. Such toxicity could be discarded by avoiding IV injection of the formulated Kolliphor^®^ HS15 at this concentration on humans.

The three studied co-surfactants Labrasol^®^, Labrafil^®^ M1944, and Labrafil^®^ M2125 are macrogolglycerides which differ by their fatty acids. From the results, it appears that only the macroglyceride with oleic acid chains, i.e., Labrafil^®^ M1944, could be envisaged safely for IV administration. This result is all the more interesting as Labrasol^®^ is a well-known solubility enhancer previously reported for the design of various poorly soluble drug-loaded nanoformulations [48], in particular, for parenteral administration. Its haemolytic activity determined at the targeted plasma concentration of 0.25 mg/mL could partly explain the mortality of some mice upon IV administration of Labrasol^®^-based nanoemulsions [35]. This result was not highlighted in previous haemolytic assays [49] but was discussed in others [47]. The haemolytic potential observed at 0.25 mg/mL renders the detrimental use of Labrasol^®^ for IV administration, although its interest in oral formulations is kept unchanged [50].

Whatever the concentration was, no haemolytic activity was highlighted for the three solvents Kollisolv^®^ PG, Kollisolv^®^ PEG 400, and Transcutol^®^ HP. These excipients that possess a high solubilizing ability are widely used in parenteral pharmaceutical formulations. Propylene glycol and PEG are commonly used, and they are recognized as safe to use for the oral, IV, and topical routes [51]. Propylene glycol is found in marketed medicines [52], and the dose limits commonly used intravenously greatly exceed those studied [53]. Transcutol^®^ HP is a high-purity solvent and solubilizer recommended for human pharmaceutical formulations administered parenterally [54]. Given these results and their attractive properties, these compounds could be incorporated into the versatile formulation to enhance the solubilization of drugs.

With regard to the haemolysis results and considering their ability to solubilize poorly soluble drugs, we have chosen 3 excipients to develop nanoemulsions compatible with IV: Labrafac^®^ WL 1349 as an oily vehicle containing medium-chains fatty acids, Kolliphor^®^ HS 15 as a surfactant, and Transcutol^®^ HP as a solubilizer. More detailed haemolytic assays defined a maximum human blood concentration of 0.5 mg/mL for Kolliphor^®^ HS 15, corresponding to an IV administrable mouse dose of 492 mg/kg (see the Supplementary Materials Figure S1). These assays confirmed the safety profiles of the chosen triglycerides and of the diethylene glycol monoethyl ether.

### 3.2. Mixture Experiments

**Figure 2:**
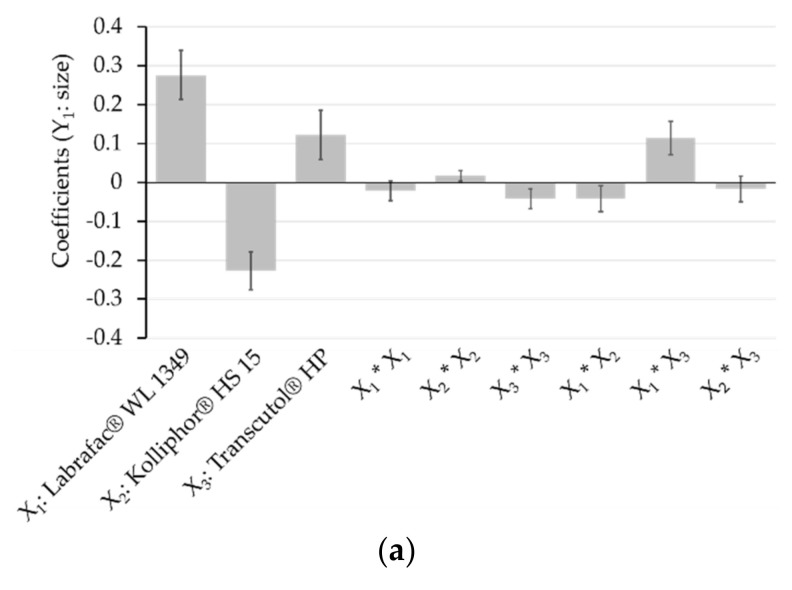

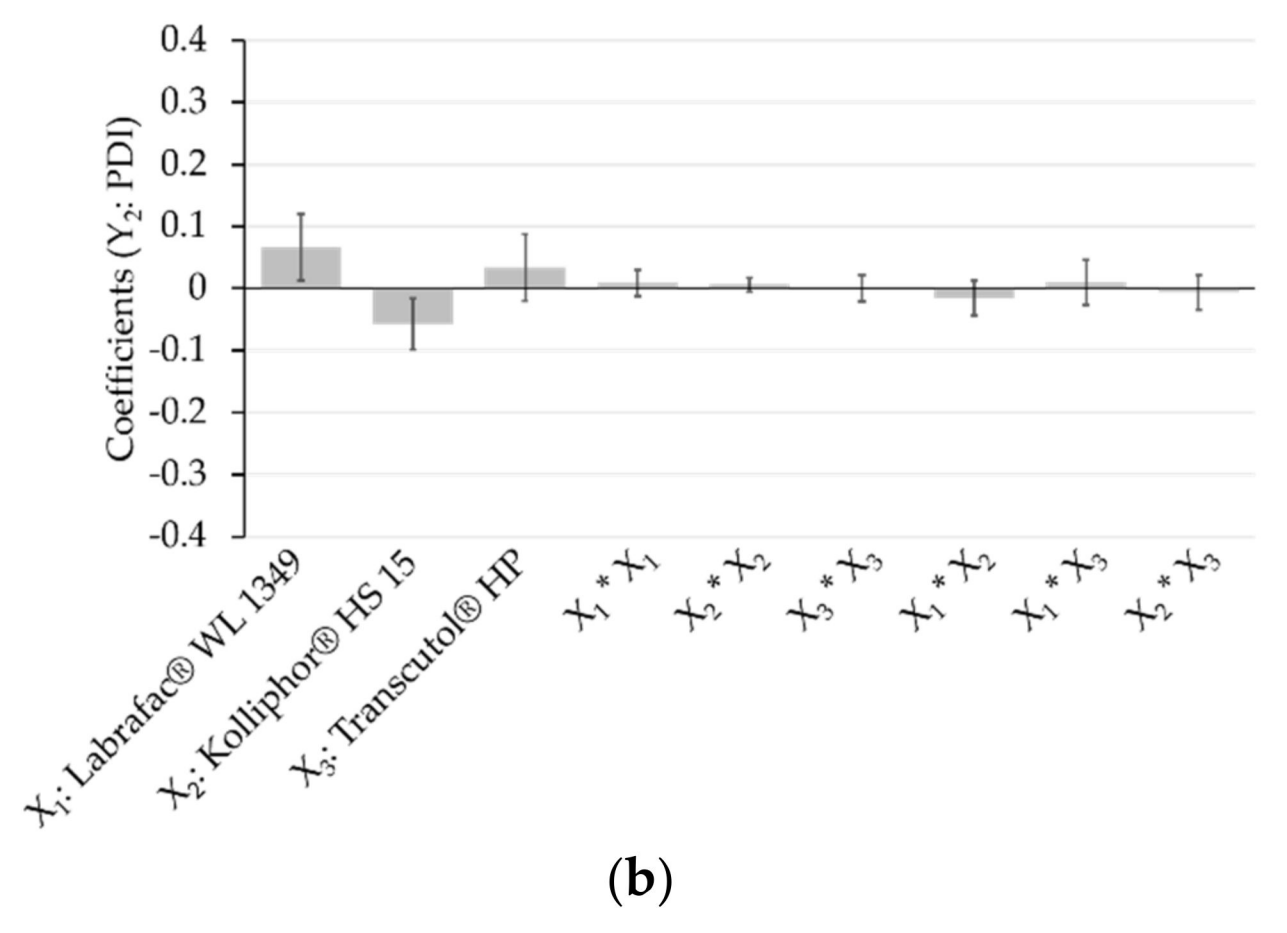
Plot of the regression coefficients of the variables X for the responses Y_1_: size (**a**) and Y_2_: polydispersity index (PDI) (**b**).

Mixture experiments were carried out to define the effects of the 3 selected excipients on the size and polydispersity of nanoemulsions formulated by transposing a spontaneous emulsification process previously established by our team [31]. The agitation rate and its duration were fixed to 750 rpm and 15 min, respectively. The design space was defined as follow (weight fractions): 0.1 < oil < 0.6, 0.3 < surfactant < 0.8, and 0.1 < solubilizer < 0.6. The water proportion being unchanged, a ternary diagram was used to plot the mixture experiments (Figure 1). Ten points, and among them, the triplicated centroid, were carried out by varying the proportions of each compound (see the Supplementary Materials Table S1). PLS regression of the two responses variables (Y_1_: average diameter; Y_2_: PDI) on the three variables studied (X_1_: Labrafac^®^ WL 1349; X_2_: Kolliphor^®^ HS 15; and X_3_: Transcutol^®^ HP) was developed according to a second-degree polynomial Cox model. If this model is valid, it is possible to interpret the influence of components on studied responses directly from coefficient values of the polynomial equation [48]. A log-transformation of the Y_1_ = diameter response simplifying the response function and making the response-X factor relationship linear was done before fitting the model. The performance statistics considered for this PLS model, performed by combining the 12 experimental points, appear as excellent for explaining the variation of Y_1_ = diameter and Y_2_ = PDI (R^2^Y_1_ = 0.983 and R^2^Y_2_ = 0.869). Whereas it is good for its cross-validated predictive ability in Y_1_ = diameter (Q^2^Y_1_ = 0.799), it appears limited for that in Y_2_ = PDI (Q^2^Y_2_ = 0.583). Indeed, R^2^ and Q^2^ should be as close to 100% as possible and preferably not be separated by more than 20–30% to point to a valid interpretable model [55]. On this basis, regression coefficients of the X variables for each Y response were plotted (Figure 2). The sizes and signs of the regression coefficients relating to centred and scaled variables indicate the contribution of each model term on the considered response. The statistical significance of each coefficient is indicated as 95% confidence intervals. The influence of the various excipients was found more significant on the diameter than on PDI, and it follows the same trend in both cases: X_1_ is the most important factor, and a higher proportion of Labrafac^®^ WL 1349 leads to larger and more polydisperse nanodroplets. Whereas the influence of the solubilizer (X_3_ = Transcutol^®^ HP) is also positive on both Y_1_ and Y_2_, the contribution of the surfactant (X_2_ = Kolliphor^®^ HS 15) enables the formation of smaller and more monodisperse colloids, as previously observed in other nanoformulations [48].

**Figure 3:**
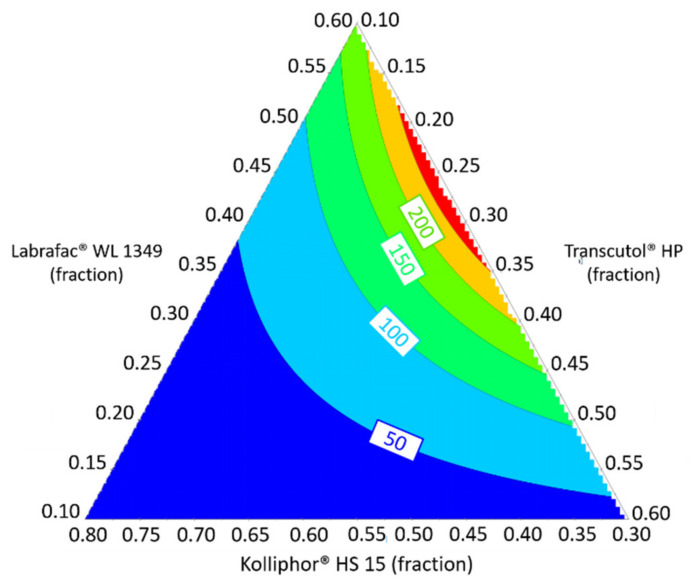
Response contour plot allowing the prediction of Y_1_: the average diameter from the regression model.

Furthermore, a mixture design was carried out in the previously defined feasibility zone, and a Cox model for response in size was fitted. The analysis of variance (ANOVA), based on F-test, was performed in order to determine the validity of the model. The F-test for the regression model indicates that it is significant at a confidence level of 95% (*p*-value = 0.00 < 0.05) and has no lack of fit at 95% (*p*-value = 0.38 > 0.05). Thus, the regression model for the size response Y_1_ appears statistically good and valid. A graphical representation of the contour plots allowing the prediction of the size is shown Figure 3. From these results, 2 compositions were determined to design nanoemulsions with average diameters of NE_1_ = 50 nm (Labrafac^®^ WL 1349/Kolliphor^®^ HS 15/Transcutol^®^ HP = 35/55/10%, *w*/*w*) and NE_2_ = 120 nm (Labrafac^®^ WL 1349/Kolliphor^®^ HS 15/Transcutol^®^ HP = 37/42/21%, *w*/*w*). These targeted diameters were chosen to avoid fast renal clearance [56], hepatocytes interactions [57], and mononuclear phagocytic system [58] in order to extend the nanoemulsion bloodstream circulation.

### 3.3. Characterization of Blank Nanoemulsions

#### 3.3.1. Droplet Size and Zeta Potential

**Figure 4:**
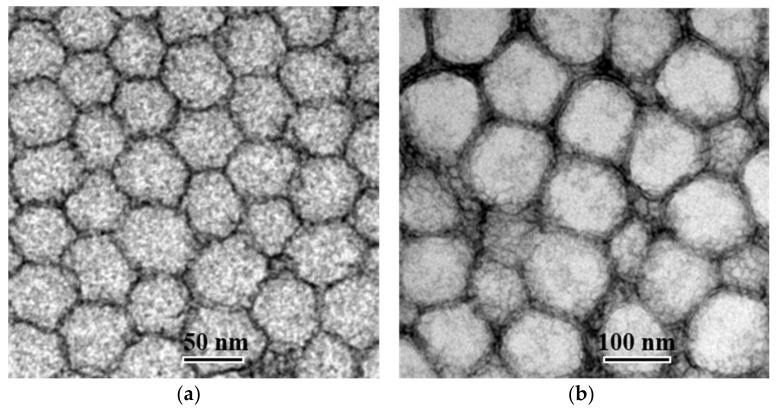
Blanks NE_1_ (**a**) and NE_2_ (**b**) under transmission electron microscopy.

Nanoemulsions were formulated by spontaneous nano-emulsification and the organic solvent-free method. Indeed, the use of organic solvents can provide safety issues and be dose-limiting due to their toxicity. DLS analysis indicated that the average particle sizes of the NE_1_ and NE_2_ were 49.0 ± 2.3 nm and 118.6 ± 0.1 nm, respectively. TEM images revealed that all droplets have a spherical shape (Figure 4), and by keeping in mind the possibilities of the TEM technique [59], sizes appear in the same range as those determined by DLS. A monodisperse population with a defined size is required to develop safe, stable, and efficient nanoparticles [60]. In the literature, various PDI acceptable limits are reported [60,61]. Nanoemulsions exhibiting PDI values below 0.2 are commonly referred to as monodisperse [62], and when the nanoparticles must be injected, PDI values up to 0.250 are needed [63]. In our case, even though the nanoemulsion polydispersity could not be predicted from a contour plot, as explained in Section 3.2.; PDI values were found to be 0.162 ± 0.027 and 0.198 ± 0.003 for NE_1_ and NE_2_, respectively, indicating a monodisperse population, suitable for IV administration. Nanoemulsions showed slightly negative zeta potential values of −7.4 ± 1.5 mV for NE_1_ and −9.4 ± 1.8 mV for NE_2_. The surface charge of nanoparticles may impact their biodistribution. Although negative zeta potential values ensure good electrochemical stability of nanoemulsions [63,64], neutral nanoparticles (±10 mV) show lower mononuclear phagocytic system uptake and the longest circulation compared to positive- or negative-charged particles [65], which is favourable to achieve efficient drug delivery after IV administration.

#### 3.3.2. Stability in Storage Conditions

**Figure 5:**
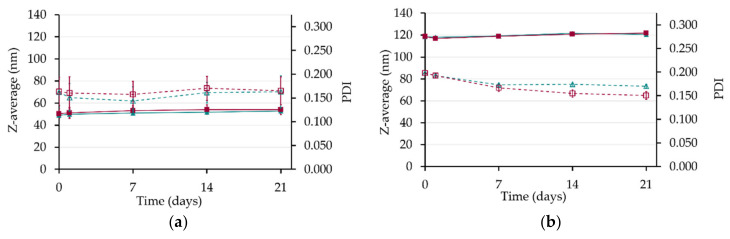
Stability of NE_1_ (**a**) and NE_2_ (**b**) estimated by the Z-average (solid line) and the PDI (dotted line) during 21-day storage at 4 °C (▲ in green) and 20 °C (■ in red).

To evaluate the nanoemulsions stability in storage conditions, they were stored undiluted at two different temperatures: 4 °C and 20 °C. Macroscopic observations, droplet diameter, and PDI were used as physical stability indicators. No visible phase separation was observed for both formulations stored at both temperatures. The absence of a significant size modification with time indicated the absence of the destabilization phenomena that usually affects nanoemulsions, i.e., droplet aggregation, coalescence, or Ostwald ripening [66]. Despite neutral values of zeta potential, these results demonstrated their good stability after a 21-day storage (Figure 5), which may be explained by the steric hindrance of PEG moiety on the surface, imparted by Kolliphor^®^ HS 15 [67]. Hence, these nanoemulsions can be stored either at cold or controlled room temperatures for a long storage period.

#### 3.3.3. Stability in Biomimetic Conditions

**Figure 6:**
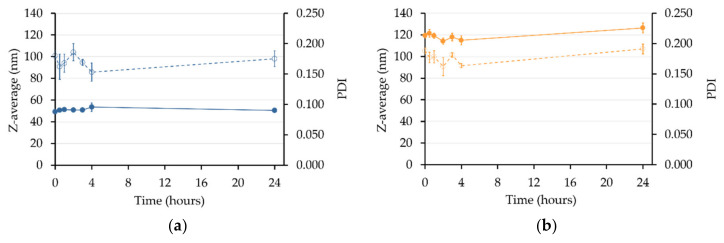
Stability of NE_1_ (**a**) and NE_2_ (**b**) at 37 °C in PBS estimated by the Z-average (●) and the PDI (○) evolution for 24 h.

Considering that nanoemulsion stability is a major issue for parenteral administration, nanoemulsions samples were prepared with a 1/100 dilution in PBS as plasma biomimetic medium at 37 °C. It is known that temperature may significantly impact the stability of nanoemulsions by modifying excipient’s properties and interfaces [63]. Both nanoemulsions remained homogenous and stable for 24 h (Figure 6), suggesting good stability of the nanoemulsions in the bloodstream. However, a major issue with nanoemulsion stability is the modification of the droplet interface by plasmatic proteins, which can lead to aggregation. Consequently, it would be interesting to study the stability of these nanoemulsions in PBS with proteins or in plasma.

#### 3.3.4. Haemolytic Profile of the Developed Nanoemulsions

Although they do not substitute in vivo tests to date, the development of in vitro tests pable to providing informations on cytotoxicity is highly recommended during nonclinical studies [68,69]. As the nanoemulsions are intended for the IV route, lack of toxicity and haemocompatibility of nanoemulsions have to be insure. Both nanoemulsions, used at the theoretical plasma concentration of 0.25 mg/mL, showed no haemolysis after 3 h of incubation with human whole blood. This concentration is interesting to consider because it could correspond to the administration of 10 mL of nanoemulsions by the IV route, which is coherent with volumes used in clinics. NE_2_ remains nontoxic whatever the concentration, whereas an increase of the Kolliphor^®^ HS 15 proportion in NE_1_, i.e., the formulation with smaller droplets, results in significant haemolysis (>5%) at 2.5 mg/mL (Table 4). Such a concentration is discarded in the clinic or would be locally decreased upon slow IV injection rate.

Besides, in order to be adapted to IV administration, pH and osmolarity must be adjusted. The pH values of NE_1_ and NE_2_ were 7.2 ± 0.1 and 7.3 ± 0.3, respectively. Nanoemulsions showed osmolarity values of 283 ± 10 mOsm for NE_1_ and 275 ± 10 mOsm for NE_2_. The pH and osmolarity values and the results of the haemolysis test suggest that the developed nanoemulsions were suitable for IV.

### 3.4. Encapsulation of Ibuprofen and Fenofibrate

#### 3.4.1. Formulation of API-Loaded Nanoemulsions

Ibuprofen and fenofibrate were selected as water practically insoluble APIs models. Ibuprofen is a well-known non-steroidal anti-inflammatory drug widely used to treat pain and inflammation. Ibuprofen, used as a water insoluble model compound, has a solubility of 3.44 mg/mL in PBS at 37 °C [70]. Fenofibrate is a very potent and highly effective lipid lowering agent used in the treatment of hypercholesterolemia. It was employed as a model chemically stable drug, which is poorly water soluble: 0.0007 mg/mL at 37 °C [71]. Increased solubility would result in improved absorption in the digestive tract. Various ways have been explored to increase the solubilization rate of fenofibrate. Nanoemulsion can be envisaged as an interesting strategy since it is reported highly soluble in lipid droplets. Ibuprofen and fenofibrate were successfully encapsulated in both nanoemulsions without protocol modification and with an encapsulation efficiency always higher than 90% for NE_1_ and at least 80% for NE_2_. Ibuprofen was incorporated into nanoemulsions from 2% to 16% (*w*/*w*) drug-loading rates (e.g., with an ibuprofen DL rate of 16%, and EE equal to 103.5 ± 3.1% and 82.6 ± 2.9% for NE_1_ and NE_2_, respectively). In the case of fenofibrate, its apparent solubility was increased more than 15,000 times thanks to an up to 10% (*w*/*w*) drug-loading rate (e.g., with a fenofibrate DL rate of 10%, and EE equal to 96.5 ± 1.6% and 84.5 ± 4.2% for NE_1_ and NE_2_, respectively). The high encapsulation efficiency (>90%) and high drug-loading rates are higher than lipid-based drug-delivery systems reported in the literature (loading capacities for ibuprofen up to 6% (*w*/*w*) [72,73,74,75] and for fenofibrate up to 7.5% (*w*/*w*) [76,77,78]). Interestingly, this high encapsulation capacity of water-insoluble APIs into the developed nanoemulsions was obtained from biocompatible and biodegradable excipients without any use of potentially toxic organic solvents.

#### 3.4.2. Characterization of API-Loaded Nanoemulsions

**Figure 7:**
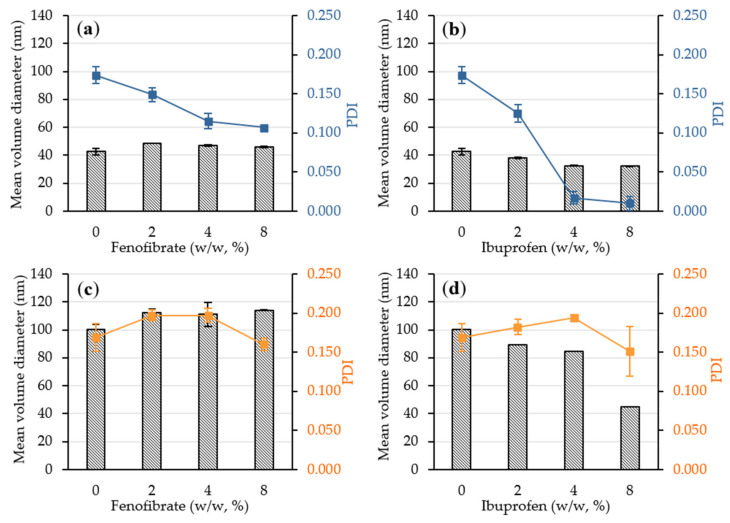
Droplet diameter in volume (bars) and PDI (solid lines) of NE_1_ (**a**,**b**) and NE_2_ (**c**,**d**) as a function of drug loading.

Unexpectedly, droplet size and PDI decrease with ibuprofen loading increase (Figure 7). Indeed, in case of NE_2_, the mean droplet diameter decreased from 100.3 nm to 45.1 nm when the ibuprofen content increased from 0 to 8% (*w*/*w*). To a lesser extent, the same phenomenon was observed for NE_1_. Although Lee et al. [79] observed the same performance of ibuprofen on droplet diameter and PDI, API loading and especially ibuprofen usually causes droplet diameter increase [80]. Ibuprofen is at least twice more soluble in Kolliphor^®^ HS 15 than in Labrafac^®^ WL 1349 and even more soluble in Transcutol^®^ HP [31,81] (i.e., solubility of 694 mg/mL [82]). By anchoring at the oil–water interfaces, ibuprofen could stabilize droplets and improve their monodispersity. As fenofibrate is equally soluble in Labrafac^®^ WL 1349 and in Kolliphor^®^ HS 15 [31], it can be integrated into the droplets corona and be well solubilized into their oily core. Conversely, the addition of fenofibrate causes a slight increase in droplet size regardless of the amount added.

#### 3.4.3. In Vitro API Release Studies

**Figure 8:**
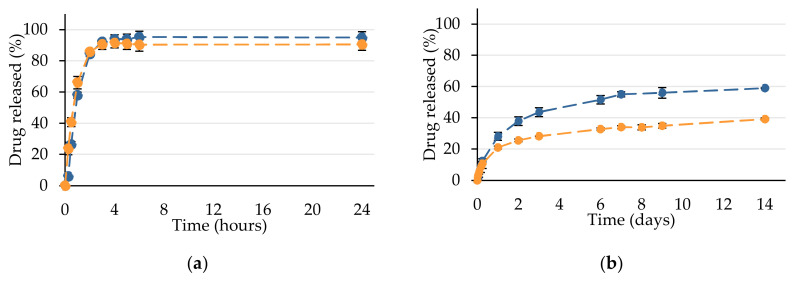
Experimental drug release kinetics in sink conditions of 2 wt% ibuprofen (**a**) and fenofibrate (**b**) loaded NE_1_ (● in blue) and NE_2_ (● in orange).

In vitro ibuprofene and fenofibrate release studies were performed on 2 wt% drug-loaded NE_1_ and NE_2_ using the dialysis bag method (Figure 8). For fenofibrate, sink conditions were assured by the addition of Tween^®^ 80 to the release medium. Indeed, an increase of the saturation solubility of fenofibrate in the presence of polysorbate 80 micelle-forming species is obtained, in particular at 37 °C [83].

Ibuprofen release appears quite rapid since, in 2 h, 85 ± 1% of the encapsulated drug was released from both nanoemulsions, independently of their initial composition and of the available droplet area. By considering these 2 first hours, it appears that ibuprofen release from NE_1_ rather follows a linear process (r^2^ = 0.9539), while it best fits into a square root of time model (r^2^ = 0.9868) from NE_2_, suggesting differences of organization of the API within the droplets.

Fenofibrate release from both NE_1_ and NE_2_ was found to follow a square root of time kinetic model with r^2^ > 0.99. As the relative affinity of the API for nanodroplets and the release medium directly influences the drug-release profile [31], fenofibrate release rate constants described by slopes of the plots are lower than those of ibuprofen (Table 4). Moreover, a faster drug release is observed in the case of fenofibrate-loaded NE_1_, in coherence with the larger interfacial area accompanying the smaller nanoemulsion droplets. Fenofibrate would be well dispersed within the nanodroplets matrix, in particular, thanks to Transcutol^®^ HP because of its highest affinity. This polar protic solubilizer demonstrates affinity and good miscibility with polar lipids asmedium chain triglycerides and polyethylene glycol-based surfactants such as those used in the present formulation [84]. Such an organization results in sustained release in 14 days of 59% of fenofibrate from the smaller NE_1_ and that of 39% from NE_2_. Even though formulation of the nanoemulsion is made below the melting temperature of the drug, i.e., 81 °C [85], fenofibrate molecules appear well solubilized within the excipients. Such a homogeneous dispersion within the excipients would permit to prevent recrystallization during its dissolution [86]. Thus, the developed nanoemulsions are very promising to favour improved fenofibrate absorption and to increase bioavailability. Such results could be transposed to other poorly soluble drugs to permit their preclinical evaluation.

## 4. Conclusions

On the basis of the biocompatibility profile of excipients assessed from a miniaturized haemolytic test and through a design of experiments, two controlled nanodroplets formulations were developed. These nanoemulsions generated from a simple self-emulsification formulation process appeared very performant to improve solubility of practically insoluble APIs without any use of organic solvents. Being storage stable with optimized physicochemical properties for IV administration and able to deliver APIs according to a modulable profile, these developed nanoemulsions appear to be very promising drug-delivery systems. Thanks to this nanoscale tool, the challenge of poor solubility could be overcome and valuable preclinical and even clinical studies of poorly soluble drugs could be more serenely envisaged.

## Figures and Tables

**Table 1:** Values of the correlation coefficients (r^2^) and drug release rate constant (k) obtained by fitting ibuprofen or fenofibrate release data from NE_1_ and NE_2_ with zero-order, first-order and square of time release mathematical models.

		Zero Order		First Order		√t	
		r^2^	k (mgh^−1^)	r^2^	k (h^−1^)	r^2^	k (mgh^−1/2^)
Ibuprofen	NE_1_	0.9539	0.823	0.7157	1.286	0.9105	1.198
	NE_2_	0.9017	0.980	0.8098	0.661	0.9868	1.530
Fenofibrate	NE_1_	0.9304	0.021	0.608	0.058	0.9917	0.158
	NE_2_	0.9112	0.022	0.7632	0.069	0.9991	0.124

**Table 2:** HPLC methods used in the present work.

API	λ (nm)	Flow (mL/min)	T (min)	Gradient T (min)	A (%)	Vinj (µL)	Rt (min)	Concentration Range (µM)
Ibuprofen	263	0.6	1.5	0	40	8.0	0.66	5–100
		0.8		0.2	60			
		0.8		0.6	98			
Fenofibrate	290	0.6	3.5	0	60	3.0	2.00	5–150
				1.5	68			
				2.2	68			
				2.3	100			

**Table 3:** Results of the haemolysis test performed on various excipients (ND: not determined; results above 5% of haemolysis have been greyed out).

		Percentage of Haemolysis (%)			
Excipient Concentration (mg/mL)		0.05	0.1	0.25	2.5
**Labrafac^®^ WL 1349**	**Medium chain triglyceride**	**<1**	**<1**	**<1**	**1.1 ± 0.8**
Miglyol^®^ 812	Medium chain triglyceride	1.2 ± 0.5	<1	1.0 ± 0.9	3.2 ± 0.9
Maisine^®^ CC	Glycerol monolinoleate	ND			
Peceol^®^	Glycerol mono-oleates (type 40)	ND			
Plurol^®^ Oleique CC 497	Polyglyceryl-3 dioleate	ND			
Lipoid^®^ E PC	Egg yolk phosphatidylcholine	3.2 ± 0.22	3.5 ± 0.9	3.8 ± 0.6	5.0 ± 0.8
Lipoid^®^ S 100	Soy phosphatidylcholine	3.0 ± 0.4	3.5 ± 1.6	3.1 ± 0.5	3.7 ± 1.0
Lipoid^®^ S LPC 80	Soy lysophosphatidylcholine	69.6 ± 7.3	ND	54.5 ± 7.8	67.7 ± 94.4
Kollisolv^®^ PEG 400	Macrogol	2.0 ± 0.6	2.0 ± 0.5	3.1 ± 1.4	2.5 ± 1.3
**Kolliphor^®^ HS15**	**Macrogol 15 hydroxystearate**	**<1**	**<1**	**1.0 ± 0.8**	**22.9 ± 15.1**
Kolliphor^®^ EL	Macrogolglycerol ricinoleate	<1	1.3 ± 3.1	<1	4.6 ± 0.2
Kollisolv^®^ PG	Propylene glycol	2.4 ± 0.9	3.4 ± 1.2	<1	<1
Captex^®^ 200	Propylene glycol dicaprylocaprate	1.6 ± 0.8	1.5 ± 0.1	2.6 ± 1.9	19.9 ± 10.4
Labrafil^®^ M1944	Oleoyl macrogol-6 glycerides	<1	ND	3.6 ± 0.9	100.0 ± 15.8
Labrafil^®^ M2125	Linoleoyl macrogol-6 glycerides	1.6 ± 0.3	3.8 ± 0.6	61.2 ± 4.1	≥100
Labrasol^®^	Caprylocaproyl macrogol-8 glycerides	<1	<1	27.1 ± 8.5	≥100
**Transcutol^®^ HP**	**Diethylene glycol monoethyl ether**	**<1**	**<1**	**<1**	**<1**
Tween^®^ 80	Polysorbate 80	1.2 ± 0.2	2.1 ± 0.5	20.9 ± 4.8	86.7 ± 2.7

**Table 4:** Results of haemolysis test for NE_1_ and NE_2_.

Nanoemulsions Concentration (mg/mL)	Percentage of Haemolysis			
	NE_1_		NE_2_	
	Average	Standard Deviation	Average	Standard Deviation
0.05	<1	NA	<1	NA
0.10	<1	NA	<1	NA
0.25	<1	NA	<1	NA
0.50	1.5	0.2	<1	NA
1.00	2.9	0.2	<1	NA
2.50	9.9	1.6	<1	NA

Abbreviations: NA, not applicable.

## Supplementary Materials

The following are available online at https://www.mdpi.com/1999-4923/12/12/1141/s1, Figure S1: Results of the haemolysis test for the chosen excipients, Table S1: Mixture design comprising 12 experiments where the central point was triplicated, Table S2: Names, structures and characteristics of tested excipients.
