# Supplementary material for: Design of Non-Haemolytic Nanoemulsions for Intravenous Administration of Hydrophobic APIs
Source: Pharmaceutics. 2020 Nov 25;12(12):1141. doi: 10.3390/pharmaceutics12121141 (PMC7760703; doi:10.3390/pharmaceutics12121141)
Supplement: Supplementary file 1 [file pharmaceutics-12-01141-s001.pdf]

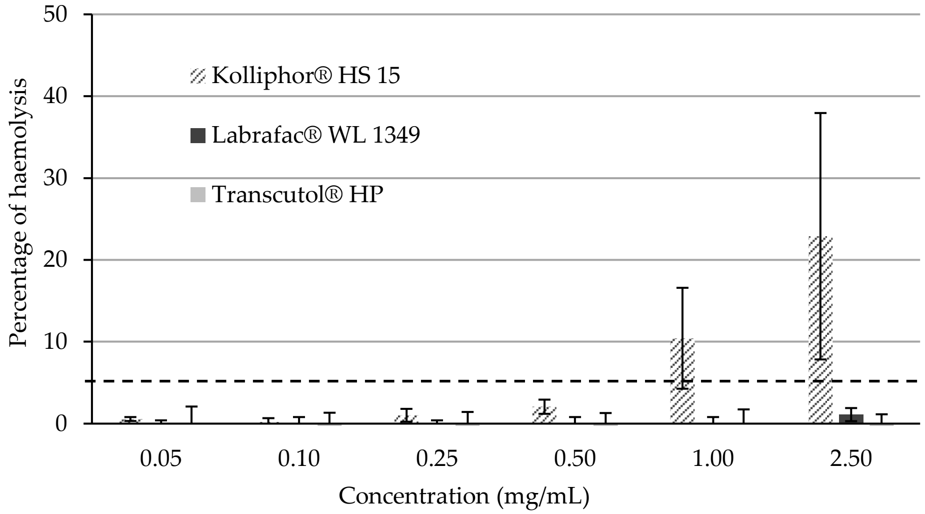

Figure S1. Results of the haemolysis test for the chosen excipients: the dotted line represents the threshold value of 5%. Note: Data are shown as mean ± standard deviation (n = 3).

Table S1. Mixture design comprising 12 experiments where the central point was triplicated.

Trial	X_1: Labrafac® WL 1349 (%, w/w)	X_2: Kolliphor® HS 15 (%, w/w)	X_3: Transcutol® HP (%, w/w)	Y_1: Average Diameter (nm)	Y_2: PDI
1	60	30	10	179.0	0.480
2	10	80	10	17.7	0.098
3	10	30	60	34.1	0.236
4	35	55	10	49.7	0.182
5	35	30	35	242.8	0.400
6	10	55	35	25.1	0.183
7	43.3	38.3	18.3	152.1	0.217
8	18.3	63.3	18.3	23.9	0.090
9	18.3	38.3	43.3	71.5	0.234
10	26.7	46.7	26.7	61.3	0.204
11	26.7	46.7	26.7	67.6	0.245
12	26.7	46.7	26.7	77.4	0.207

Table S2. Names, structures and characteristics of tested excipients

Trade name	Denomination	Molecular structure	Typical properties
**Labrafac® WL 1349**	Medium chain triglyceride<br>*Fatty acid composition: Caprylic acid (50 to 80%) and capric acid (20 to 50%)*	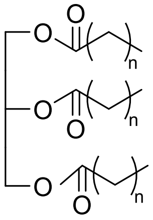 n = 6-8<br>**Glycerides, mixed decanoyl and octanoyl**	• MW = 512 g/mol<br>• HLB = 1
**Miglyol® 812 N**	Medium chain triglyceride<br>*Fatty acid composition: Caprylic acid (50 to 65%) and capric acid (30 to 45%)*	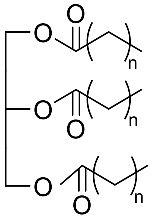 n = 6-8<br>**Glycerides, mixed decanoyl and octanoyl**	• HLB = 1
**Maisine® CC**	Glycerol monolinoleate<br>*Fatty acid composition: Linoleic acid (≥50%), oleic acid (10 to 35%), palmitic acid (4 to 20%), stearic acid (≤6%) and traces of linolenic acid, arachidic acid and eicosenoic acid*	Monoglyceride (32 to 52%): 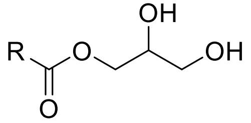<br>Diglyceride (40 to 55%): 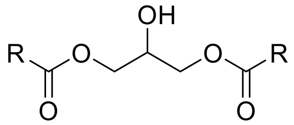<br>Triglyceride (5 to 20%): 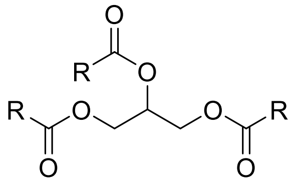<br>R = 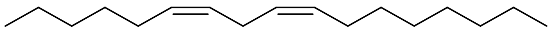 or 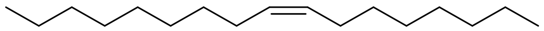<br>**Mixture of mono-, di- and triglycerides of oleic and linoleic acid (C18 :1/C18:2)**	• HLB = 1

**Peceol®**	Glycerol mono-oleate (type 40)<br><br>***Fatty acid composition: Oleic acid (≥60%), linoleic acid (≤35%), palmitic acid (≤12%), stearic acid (≤6%) and traces of arachidic acid, eicosenoic acid and linolenic acid***	Monoglyceride (32 to 52%): 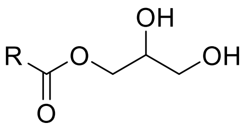<br>Diglyceride (30 to 50%): 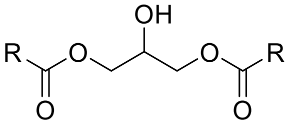<br>Triglyceride (5 to 20%): 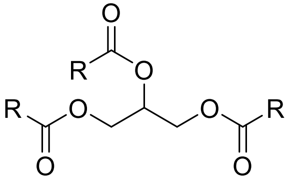<br>R = 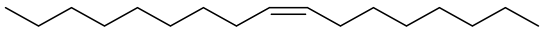<br>**Mixture of mono-, di- and triglycerides of oleic acid (C18 :1)**	• MW = 356.55 g/mol<br>• HLB = 1
**Plurol® Oléique CC 497**	Polyglycerol-3 dioleate<br><br>***Fatty acid composition : Oleic acid (65 to 88%), linoleic acid (5 to 18%), palmitic acid (2 to 16%) and traces of linolenic acid, myristic acid, palmitoleic acid and stearic acid***	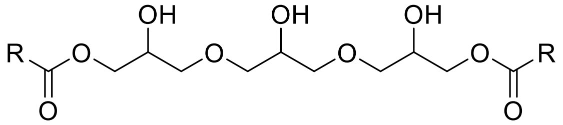<br>R = 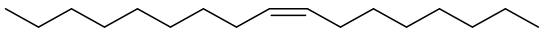<br>**Polyglycerol-3 esters of oleic acid (C18 :1)**<br>(Mixture of mono-, di-, tri-, tetra- and pentaesters of triglycérol)	• HLB = 3
**Lipoid® E PC**	Egg yolk phosphatidylcholine<br><br>***Fatty acid composition: Palmitic acid (33%), oleic acid (27%), linoleic acid (17%), stearic acid (14%), arachidonic acid (4%), and palmitoleic acid (1%)***	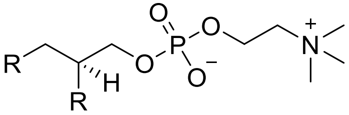<br>**Phosphatidylcholine**<br>R =<br>Palmitic acid (C16:0) 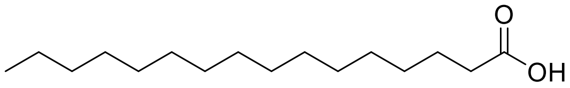<br>Oleic acid (C18:1) 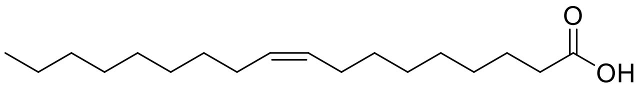<br>Linoleic acid (C18:2) 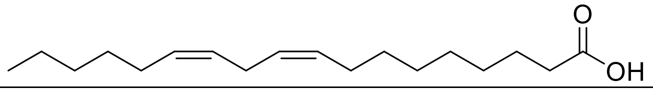	• MW = 775 g/mol<br>• HLB ≈ 6.58

**Lipoid® S 100**	Soy phosphatidylcholine<br><br>*Fatty acid composition: Linoleic acid (62%), palmitic acid (15%), oleic acid (12%), linolenic acid (5%), and stearic acid (3%)*	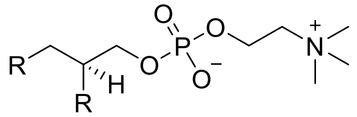<br>**Phosphatidylcholine**<br>R =<br>Palmitic acid (C16:0) 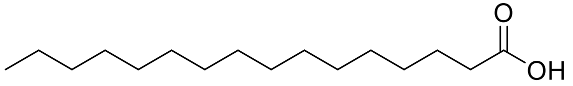<br>Oleic acid (C18:1) 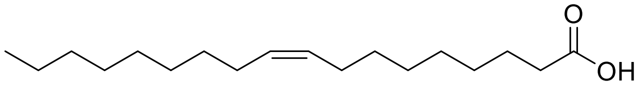<br>Linoleic acid (C18:2) 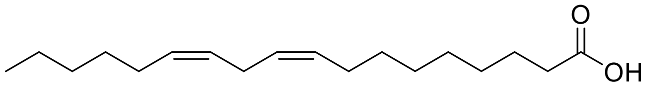	• MW = 786 g/mol<br>• HLB ≈ 6.49
**Lipoid® S LPC 80**	Soy lysophosphatidylcholine<br><br>*MAPC (80%), Phophatidylcholine (13%) with various fatty acid: linoleic, palmitic, stearic, and oleic acids*	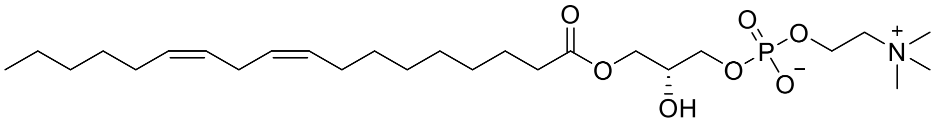<br>**Monoacyl phosphatidylcholine (MAPC)**	• MW ≈ 522 g/mol<br>• HLB ≈ 9.77
**Kollisolv® PEG 400**	Polyethylene glycol (PEG)	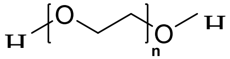<br>**Polyethylene glycol**	• MW = 400 g/mol<br>• HLB = 14
**Kolliphor® HS15**	Macrogol 15 hydroxystearate<br><br>*70% of polyglycol mono- and di-esters of 12-hydroxystearic acid (PEG 660), 30% of free PEG*	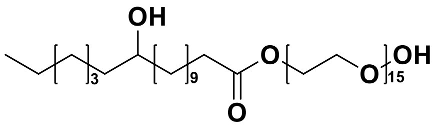<br>**Polyethylene glycol 660 12-hydroxystearate**	• MW = 911 g/mol<br>• HLB = 14-16<br>• CMC = 0.005-0.02%
**Kolliphor® EL**	Macrogolglycerol ricinoleate	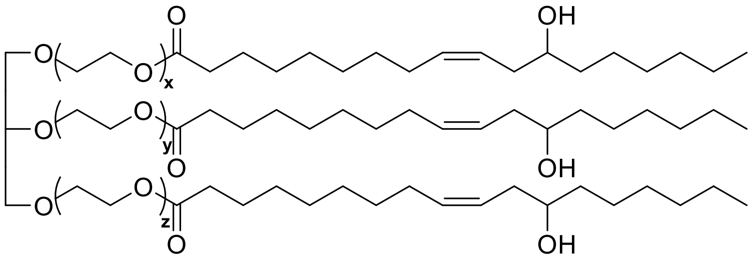 x + y + z = 35	• MW ≈ 2500 g/mol<br>• HLB = 12-14<br>• CMC = 0.2% w/w at 37°C
**Kollisolv® PG**	Propylene glycol	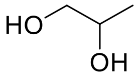<br>**1,2 Propandiol**	• MW = 76.09 g/mol

**Captex® 200**	Propylene glycol dicaprylocaprate	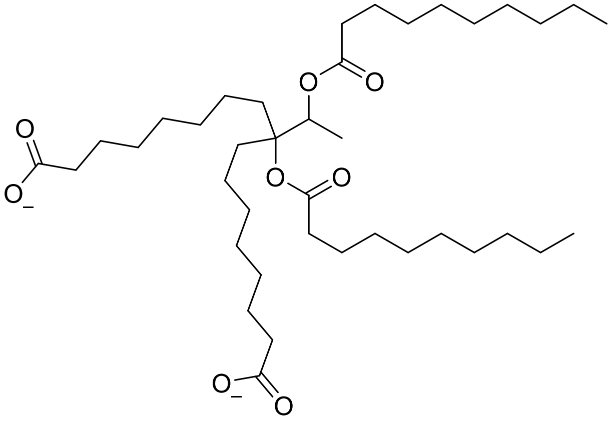<br>**Mixed diesters of caprylic/capric acids of propylene glycol**	
**Labrafil® M 1944**	Oleoyl macrogol-6 glycerides<br>*Fatty acid composition: Oleic acid (58 to 80%), linoleic acid (15 to 35%), palmitic acid (4 to 9%) and traces of arachidic acid, eicosenoic acid, linolenic acid and stearic acid*	Monoglyceride: 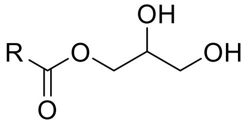<br>Diglyceride: 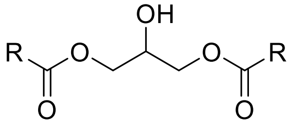<br>Triglyceride: 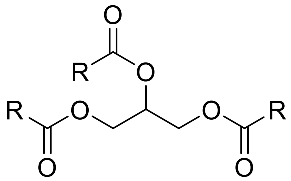<br>PEG_{300} monoester: 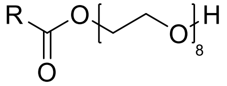<br>PEG_{300} diester: 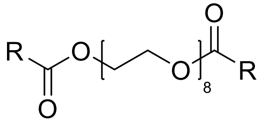<br>R = 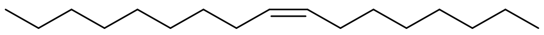<br>**Mono-, di- and triglycerides and mono- and di- PEG-6 esters of oleic acid (C18 :1)**	• HLB = 3 or 9 (according to the considered source)

**Labrafil® M 2125**	Linoleoyl macrogol-6 glycerides<br><br>*Fatty acid composition: Linoleic acid (50 to 65%), oleic acid (20 to 35%), palmitic acid (4 to 20%) and traces of arachidic acid, eicosenoic acid, linolenic acid and stearic acid*	Monoglyceride: 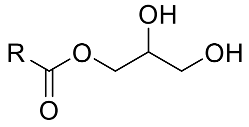<br>Diglyceride: 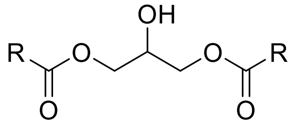<br>Triglyceride: 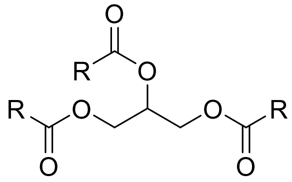<br>PEG_{300} monoester: 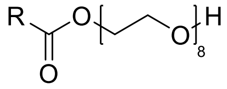<br>PEG_{300} diester: 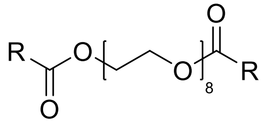<br>R = 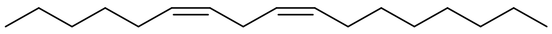<br>**Mono-, di- and triglycerides and mono- and di- PEG-6 esters of linoleic acid (C18 :2)**	• HLB = 3 or 9 (according to the considered source)

**Labrasol®**	Caprylocaproyl macrogol-8 glycerides<br><br>*Fatty acid composition: Caprylic acid (50 to 80%), capric acid (20 to 50%), and traces of caproic acid, lauric acid and myristic acid*	Monoglyceride : 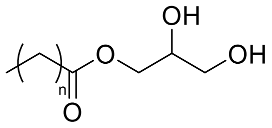<br>Diglyceride: 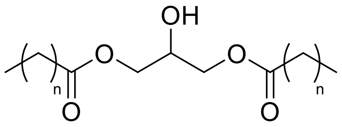 n = 6-8<br>Triglyceride: 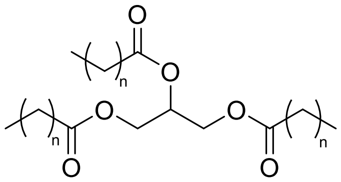<br>PEG400 monoester: 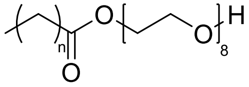<br>n = 6-8<br>PEG400 diester: 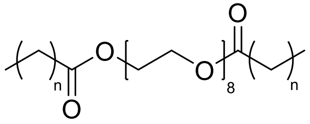<br>**Mono-, di- and triglycerides of Caprilyc/Capric acids ($C_8 + C_{10}$) and PEG-8 mono- and di- esters of Caprilyc/Capric acids ($C_8 + C_{10}$)**	• HLB = 12<br>• CMC = 0.01% (v/v) at 37°C
**Transcutol® HP**	Diethylene glycol monoethyl ether	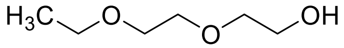<br>**2-(2-Ethoxyethoxy)ethanol**	• MW = 134.17 g/mol
**Tween 80®**	Polysorbate 80<br><br>*Fatty acid composition: Oleic acid (≤58%), linoleic acid (18%), palmitic acid (16%), palmitoleic acid (8%), stearic acid (6%), and traces of myristic acid and linolenic acid*	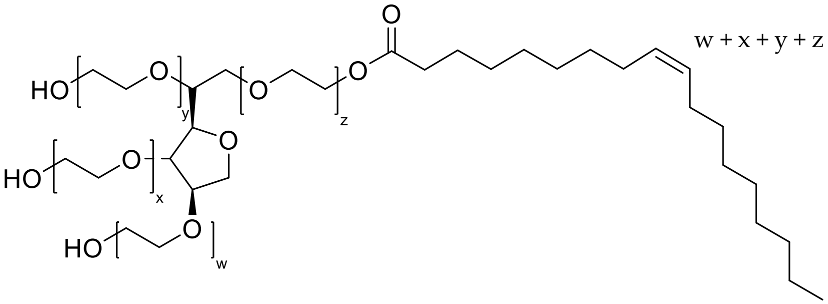<br>w + x + y + z = 20<br>**Polyoxyethylene 20 sorbitan monooleate**	• MW = 1310 g/mol<br>• HLB = 15<br>• CMC = 0.01 mM

Notes: The information was obtained from the manufacturers (BASF, Gattefosé, Lipoid GmBH, IOI Oleochemical and Abitec Corporation) and from: Jannin, *et al*., *Int. J. Pharm.* **2014**, *466*, 109-121; Koga, *et al*., *Eur. J. Pharm. Biopharm.* **2006**, *64,* 82-91; Rahali *et al*., *J. Chem. Pharm. Res.* **2014**, *6*, 1543-1547; Rowe et al., *Handbook of Pharmaceutical Excipients*; Tran et al., *Int. J. Pharm.* **2016**, *502*, 151-160; Tran, et al., *Eur. J. Pharm. Sci.* **2016**, *108*, 62-70.
